# External validation of triage tools for adults with suspected COVID-19 in a middle-income setting: an observational cohort study

**DOI:** 10.1136/emermed-2022-212827

**Published:** 2023-05-22

**Authors:** Carl Marincowitz, Laura Sbaffi, Madina Hasan, Peter Hodkinson, David McAlpine, Gordon Fuller, Steve Goodacre, Peter A Bath, Yasein Omer, Lee A Wallis

**Affiliations:** 1 Centre for Urgent and Emergency Care Research (CURE), School of Health and Related Research (ScHARR), The University of Sheffield, Sheffield, UK; 2 Information School, The University of Sheffield, Sheffield, UK; 3 Division of Emergency Medicine, University of Cape Town Faculty of Health Sciences, Cape Town, South Africa

**Keywords:** COVID-19, risk management, triage

## Abstract

**Background:**

Tools proposed to triage ED acuity in suspected COVID-19 were derived and validated in higher income settings during early waves of the pandemic. We estimated the accuracy of seven risk-stratification tools recommended to predict severe illness in the Western Cape, South Africa.

**Methods:**

An observational cohort study using routinely collected data from EDs across the Western Cape, from 27 August 2020 to 11 March 2022, was conducted to assess the performance of the PRIEST (Pandemic Respiratory Infection Emergency System Triage) tool, NEWS2 (National Early Warning Score, version 2), TEWS (Triage Early Warning Score), the WHO algorithm, CRB-65, Quick COVID-19 Severity Index and PMEWS (Pandemic Medical Early Warning Score) in suspected COVID-19. The primary outcome was intubation or non-invasive ventilation, death or intensive care unit admission at 30 days.

**Results:**

Of the 446 084 patients, 15 397 (3.45%, 95% CI 34% to 35.1%) experienced the primary outcome. Clinical decision-making for inpatient admission achieved a sensitivity of 0.77 (95% CI 0.76 to 0.78), specificity of 0.88 (95% CI 0.87 to 0.88) and the negative predictive value (NPV) of 0.99 (95% CI 0.99 to 0.99). NEWS2, PMEWS and PRIEST scores achieved good estimated discrimination (C-statistic 0.79 to 0.82) and identified patients at risk of adverse outcomes at recommended cut-offs with moderate sensitivity (>0.8) and specificity ranging from 0.41 to 0.64. Use of the tools at recommended thresholds would have more than doubled admissions, with only a 0.01% reduction in false negative triage.

**Conclusion:**

No risk score outperformed existing clinical decision-making in determining the need for inpatient admission based on prediction of the primary outcome in this setting. Use of the PRIEST score at a threshold of one point higher than the previously recommended best approximated existing clinical accuracy.

**WHAT IS ALREADY KNOWN ON THIS TOPIC**

- Uneven vaccination in low-income and middle-income settings coupled with less resilient healthcare provision means that emergency healthcare systems in low-income and middle-income countries (LMICs) may still be at risk of being overwhelmed during periods of increased COVID infection.
- Risk-stratification tools, including the PRIEST (Pandemic Respiratory Infection Emergency System Triage) score, have demonstrated accurate prediction of adverse outcomes in patients with suspected COVID-19 in high-income settings during early waves of the pandemic.
- Validation in LMICs is required if risk-stratification tools are to be used in these settings.

**WHAT THIS STUDY ADDS**

- NEWS2 (National Early Warning Score, version 2), PMEWS (Pandemic Medical Early Warning Score) and PRIEST risk-stratification tools achieved good estimated discrimination (C-statistic 0.79 to 0.82) with respect to death or organ support in the LMIC setting of the Western Cape, South Africa.

**HOW THIS STUDY MIGHT AFFECT RESEARCH, PRACTICE OR POLICY**

- Use of NEWS2, PMEWS and PRIEST at previously recommended thresholds to guide the need for hospital admission would result in a large increase in the proportion of admitted patients with marginal gains in reduced likelihood of false negative triage.
- No risk score outperformed existing clinical discharge decision-making in this setting.

## Background

The development of effective vaccines and the emergence of the clinically less severe Omicron variant means severe illness due to COVID is less common. However, uneven vaccination in low-income and middle-income settings coupled with international relaxation of COVID restrictions and less resilient healthcare provision means that emergency healthcare systems in low-income and middle-income countries (LMICs) may still be at risk of being overwhelmed during periods of increased infection.^1^

In LMICs, disposition decision-making is often based on clinician experience and gestalt.^2^ Use of risk-stratification tools to allow rapid triage of patient acuity and the need for hospitalisation can help prevent hospitals from being overwhelmed and assist less experienced clinicians in ensuring those at risk of deterioration receive inpatient treatment (or at least fair resource allocation). For application in low-income and middle-income settings, as rapid COVID tests are not available, triage tools must be applied to patients with suspected COVID and must use easily obtainable clinical factors, as opposed to laboratory and other investigations.^1 3^ Prognostic research to date has largely been conducted in inpatient and high-income settings on patients with confirmed COVID-19.^3^

The COVID-specific Pandemic Respiratory Infection Emergency System Triage (PRIEST) score and Quick COVID-19 Severity Index have shown good prediction of adverse outcomes and are included in the American College of Emergency Physicians guidance for the risk-stratification and selection of patients with suspected COVID-19 for discharge in the ED.^4–6^ The Royal College of Physicians National Early Warning Score, version 2 (NEWS2) and similar Triage Early Warning Score (TEWS) are physiology-based scores used routinely to triage patient acuity in the ED setting in the UK and South Africa.^7 8^ The WHO decision-making algorithm for respiratory infection and CRB-65 are used to risk-stratify patients with bacterial pneumonia, and Pandemic Medical Early Warning Score (PMEWS) is used in patients with influenza.^9–11^ However, validation of the accuracy and potential use of such risk-stratification tools to select patients for discharge from the ED in low-income and middle-income settings in COVID-19 has been limited, as has validation in the Omicron wave.^3^ No previous studies have compared the performance of risk scores to clinical discharge decision-making in the ED for patients with suspected COVID in this setting.

Our study aimed to

1. Validate available risk-stratification scores in adults with suspected COVID-19 infection in the Western Cape Province of South Africa (a middle-income setting).
2. Assess the accuracy of risk-stratification scores during the Omicron wave.
3. Compare accuracy of risk-stratification scores to existing clinical decision-making.

## Methods

### Study design

This retrospective observational cohort study used routinely collected clinical electronic healthcare data from EDs across the Western Cape, from the Hospital Emergency Centre Triage and Information System (HECTIS)^12^ data repository to assess the accuracy in the ED of seven clinical risk-stratification tools (PRIEST tool, Quick COVID-19 Severity Index, TEWS, NEWS2, WHO algorithm, CRB-65 and PMEWS) recommended for use in hospitalised patients with COVID-19 or similar respiratory infections (triage tools shown in online supplemental material 1).^4 5 8 9 13–15^ The study was conducted and reported in accordance with Reporting of studies Conducted using Observational Routinely-collected Data (RECORD) guidelines.^16^

10.1136/emermed-2022-212827.supp1 Supplementary data

### Setting

We obtained data from patients with suspected COVID-19 infection who attended public sector EDs in the Western Cape Province. This is one of the nine provinces in South Africa and has almost 7 million inhabitants, of whom three-quarters use public sector services.^17^ South Africa is classified by the United Nations as an upper middle-income country based on its gross domestic product per capita.^18^ A convenience sample (based on those hospitals using the recently implemented HECTIS system) was selected of seven hospital EDs, representing predominantly urban, Cape Town metropole district and regional hospitals, as well as a large peri-rural hospital ED. Clinical decision-making around patients with COVID was largely based on clinician gestalt and experience, contextualised to the local and hospital status, that is, at times hospitals were overwhelmed with COVID admissions and admission thresholds raised.^19^ Although there were intensive care unit (ICU) admission tools developed and applied,^20^ there were no specific prognostic or disposition tools applied in the ED beyond routine triage with South African Triage Scale (SATS).

### Data sources and linkage

Data on ED clinical presentation are routinely collected by the HECTIS system, including presenting complaint, triage variables (using SATS which includes TEWS) and outcome of ED consultation. Through a deterministic matching based on unique patient hospital numbers (performed by the Western Cape Provincial Health Data Centre (PHDC)),^17^ linked data were obtained which included COVID test results from the National Health Laboratory Services, comorbidities (based on prior health system encounters, including chronic prescriptions), data around admissions and movements within the healthcare system during the index COVID encounter and death (if within, or reported to, the healthcare system). For patients with multiple ED attendances, data were extracted from the initial triage data collected for the first ED attendance and outcomes were assessed up to 30 days from this index attendance.

### Inclusion criteria

Our final cohort consisted of all adults (aged 16 years and over) at time of first (index) ED attendance between 27 August 2020 and 11 March 2022, where a clinical impression of suspected or confirmed COVID-19 infection had been recorded on the HECTIS system. This time period included several waves of COVID-19 infection in South Africa, each designated by the responsible variant, comprising the Alpha (March 2020 to September 2020), Beta (November 2020 to February 2021), Delta (May 2021 to October 2021) and Omicron (November 2021 to February 2022).^21^ For those with multiple presentations during the study period, analysis was limited to the index presentation.

### Outcome

The primary composite outcome was intubation or non-invasive ventilation in the ED on index attendance, ICU admission or inpatient death up to 30 days from ED index attendance.

The secondary outcomes were inpatient death and ICU admission up to 30 days from index ED attendance.

### Patient characteristics

Initial physiological parameters and presenting complaints at triage during the patients first (index) presentation to the emergency centre were extracted from the HECTIS database. Where no comorbidities were found, they were assumed not to be present. Implausible physiological variables were set to missing as follows: systolic blood pressure <50 mm Hg, temperature >42°C or <25°C, heart rate <10/min, oxygen saturation <10% and respiratory rate=0/min.

### Analysis

We retrospectively applied the seven triage tools to our cohort to assess their accuracy for the primary and secondary outcomes.^4 5 8 9 13–15^ Online supplemental material 1 provides details of scoring and handling missing data for the triage tools. For each tool, we plotted the receiver operating characteristic (ROC) curve and calculated the area under the ROC curve (C-statistic) for discriminating between patients with and without adverse outcome. We calculated sensitivity (proportion of true positives identified, used to rule out), specificity (proportion of true negatives identified, used to rule in), positive predictive value (PPV) and negative predictive value (NPV) at the following prespecified decision-making thresholds based on recommended or usual use: 0 vs 1+ CRB-65; 0–1 vs 2+ NEWS2; 0–2 vs 3+ PMEWS; 0–4 vs 5+ PRIEST; 0 vs 1+ WHO score; TEWS 0–2 vs 2+; and Quick COVID-19 Severity Index 0–3 vs 4+.^4 7 9 22^ These tools were compared with the sensitivity, specificity, PPV and NPV of decision to admit patients to hospital on index ED attendance. Analysis was conducted for both the whole study population and the subset of patients who presented during the Omicron wave (ie, patients who presented after 31 October 2021). As previously recommended score thresholds to indicate the need for admission were based on performance in higher-income settings, sensitivity, specificity, NPV and PPV for the primary and secondary outcomes were also calculated for all other possible score cut-offs for comparison of accuracy of classification at different thresholds. Calibration slopes were plotted of expected versus observed probabilities by decile for the primary outcome for each triage score to assess agreement between observed outcomes and predictions (calibration).^23^ All analyses were performed in SPSS V.26, Python V.3.8.8 and STATA V.17.^24^

### Sample size

The sample size was fixed based on a census sample of patients with suspected COVID in the Western Cape recorded on the HECTIS during the study period.

We a priori assessed the estimated precision of the area under the ROC curve (AUC) based on a likely 5% event rate for a minimum estimated cohort of 6000 patients. Assuming an AUC of 0.8, based on previous triage tool validation studies,^22^ this sample size would provide an acceptable 95% CI width of 0.06 (95% CI 0.77 to 0.83). Our cohort size greatly exceeded the minimum sample size required for the a priori desired precision. The reported confidence interval widths indicate the range in which estimates of accuracy are likely to reside.

### Patient and public involvement

A community advisory board (CAB) was established in advance of the study, comprising eight community members affected by COVID (infected themselves or immediate family infected/hospitalised). CAB members were recruited by an experienced community liaison officer with links to key community groups. Members were intentionally sought to be representative of the various population groups and demographics of the population. The CAB discussed the ethical implications of using the routinely collected anonymised data for research and this supported the study ethics application. Through several (online) meetings, study findings were discussed with the CAB, including acceptable thresholds for clinical use of triage scores. Members had the opportunity to assess any study outputs planned for public dissemination. Due to the nature of study, the CAB were not involved in the recruitment of participants or analysis.

## Results

### Study population

**Figure 1:**
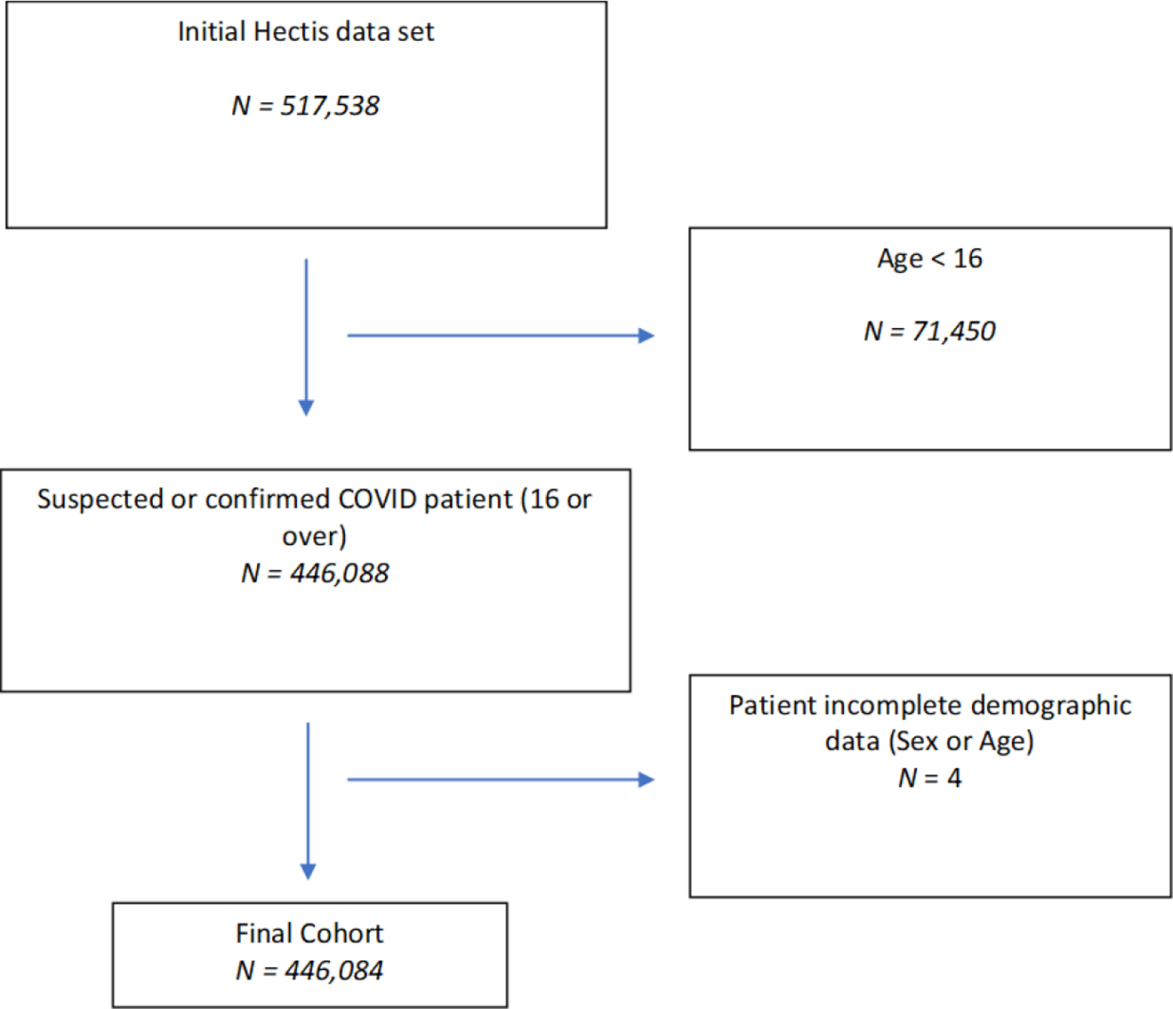
Flow diagram of study population selection.

Figure 1 and table 1 summarise the study cohort derivation and the characteristics of the 446 084 included patients. In total, 15 397 (3.45%, 95% CI 3.4% to 3.51%) experienced the primary outcome (death, ED intubation/non-invasive ventilation or ICU admission), 11 142 (2.5%, 95% CI 2.45% to 2.54%) experienced the secondary outcome of death and 2024 (0.49%, 95% CI 0.47% to 0.52%) experienced the secondary outcome of ICU admission. The Omicron period included 140 520 patients. Of these, 2787 (1.98%, 95% CI 1.91% to 2.06%) experienced the primary outcome, 1431 (1.02%, 95% CI 0.97% to 1.07%) experienced the secondary outcome of death and 677 (0.48%, 95% CI 0.45% to 0.52%) experienced the secondary outcome of ICU admission.

**Table 1:** Patient characteristics by outcome

Characteristic	Statistic/level	Adverse outcome	No adverse outcome	Total
Age (years)	N	15 397 (3.45%)	430 687 (96.55%)	446 084
	Mean (SD)	55.1 (18)	43.2 (17.1)	43.6 (17.2)
	Median (IQR)	57 (41, 69)	40 (29, 56)	41 (29, 57)
	Range	16 to 105	16 to 110	16 to 110
Sex	Male	8328 (54.1%)	221 350 (51.4%)	229 678 (51.5%)
	Female	7069 (45.9%)	209 337 (48.6%)	216 406 (48.5%)
Comorbidities	Asthma/Chronic obstructive pulmonary disease (COPD)	2694 (17.5%)	63 345 (14.7%)	66 039 (14.8%)
	Other chronic respiratory disease	76 (0.5%)	946 (0.2%)	1022 (0.2%)
	Diabetes	6057 (39.3%)	72 929 (16.9%)	78 986 (17.7%)
	Hypertension	6871 (44.6%)	116 326 (27%)	123 197 (27.6%)
	Immunosuppression (HIV)	2041 (13.3%)	75 254 (17.5%)	77 295 (17.3%)
	Heart disease	5472 (35.5%)	77 742 (18.1%)	83 214 (18.7%)
	Pregnant	105 (0.7%)	2642 (0.6%)	2747 (0.6%)
Conscious Level	Missing	652 (4.2%)	11 826 (2.8%)	12 478 (2.8%)
	Alert	10 861 (70.5%)	389 797 (90.5%)	400 658 (89.8%)
	Voice	384 (2.5%)	5598 (1.3%)	5982 (1.3%)
	Confused	797 (5.2%)	16 988 (3.9%)	17 785 (4%)
	Pain	794 (5.2%)	3177 (0.7%)	3971 (0.9%)
	Unresponsive	1909 (12.4%)	3301 (0.8%)	5210 (1.2%)
Systolic BP (mm Hg)	Missing			13 784 (3.2%)
	N	14 489	417 499	431 988
	Mean (SD)	120 (29.5)	131.2 (25.4)	131.1 (25.6)
	Median (IQR)	127 (110, 146)	128 (115, 144)	128 (115, 144)
	Range	50–289	50–300	50–300
Pulse rate (beats/min)	Missing			13 577 (3%)
	N	14 552	417 955	432 507
	Mean (SD)	99 (23.6)	93.4 (21)	93.5 (21.1)
	Median (IQR)	98 (83, 114)	92 (79, 106)	92 (79, 107)
	Range	11–300	10–300	10–300
Respiratory rate (breaths/min)	Missing			13 540 (3%)
	N	14 540	418 004	432 544
	Mean (SD)	21.7 (6.5)	18.5 (4)	18.6 (4.1)
	Median (IQR)	20 (18, 24)	18 (16, 20)	18 (16, 20)
	Range	2–60	1–60	1–60
Oxygen saturation	Missing			27 781 (6.2%)
	N	14 275	404 028	418 303
	Mean (SD)	90.5 (11.5)	96.3 (5.3)	96.1 (5.7)
	Median (IQR)	95 (87, 98)	97 (96, 99)	97 (95, 99)
	Range	10–100	10–100	10–100
Oxygen administration	Missing			26 704 (6%)
	1 (air)	7770 (53.9%)	377 447 (93.2%)	385 217 (91.9%)
	2 (40% O_2_)	404 (2.8%)	7767 (1.9%)	8171 (2%)
	3 (28% O_2_)	10 (0.1%)	304 (0.08%)	314 (0.1%)
	4 (Nasal prongs)	1242 (8.6%)	10 999 (2.7%)	12 241 (2.9%)
	5 (FM neb)	38 (0.3%)	949 (0.2%)	987 (0.24%)
	6 (rebreather mask)	1648 (11.4%)	6514 (1.6%)	8162 (2%)
	7 (nasal prongs and rebreather mask)	385 (2.67%)	996 (0.25%)	1381 (0.3%)
	8 intubated	2693 (18.7%)	0	2693 (0.6%)
	9 NIV	218 (1.5%)	0	218 (0.1%)
Temperature (°C)	Missing			12 510 (2.8%)
	N	14 743	418 831	433 574
	Mean (SD)	36.4 (1.3)	36.3 (0.8)	36.3 (0.8)
	Median (IQR)	36.4 (35.9, 37)	36.3 (36, 36.7)	36.3 (36, 36.7)
	Range	25–41.6	25–42	25–42
Cough	Missing			135 488 (30.4%)
	Present	637 (8.2%)	12 038 (4%)	12 675 (4.1%)
Fever	Missing			135 488 (30.4%)
	Present	203 (2.6%)	3998 (1.3%)	4201 (1.4%)
COVID PCR	Positive	13 027 (84.6%)	90 157 (20.9%)	103 184 (23.1%)
Hospital admission	ICU	2204 (14.3%)	0	2204 (0.5%)
Death	Within 30 days contact	11 142 (72.4%)	0	11 142 (2.5%)

ICU, intensive care unit.

In the total cohort of 446 084 patients, 65 657 (14.7%) were admitted as inpatients on index attendance. Of those, 11 862 (18.09%) experienced the primary adverse outcome. Of those not admitted on index attendance, 3535 (0.9%) experienced the primary outcome. In total, 103 184 patients (23.1%, 95% CI 23.01% to 23.23%) had a diagnosis of COVID confirmed by PCR testing at a public hospital.

### Triage tool performance

**Figure 2:**
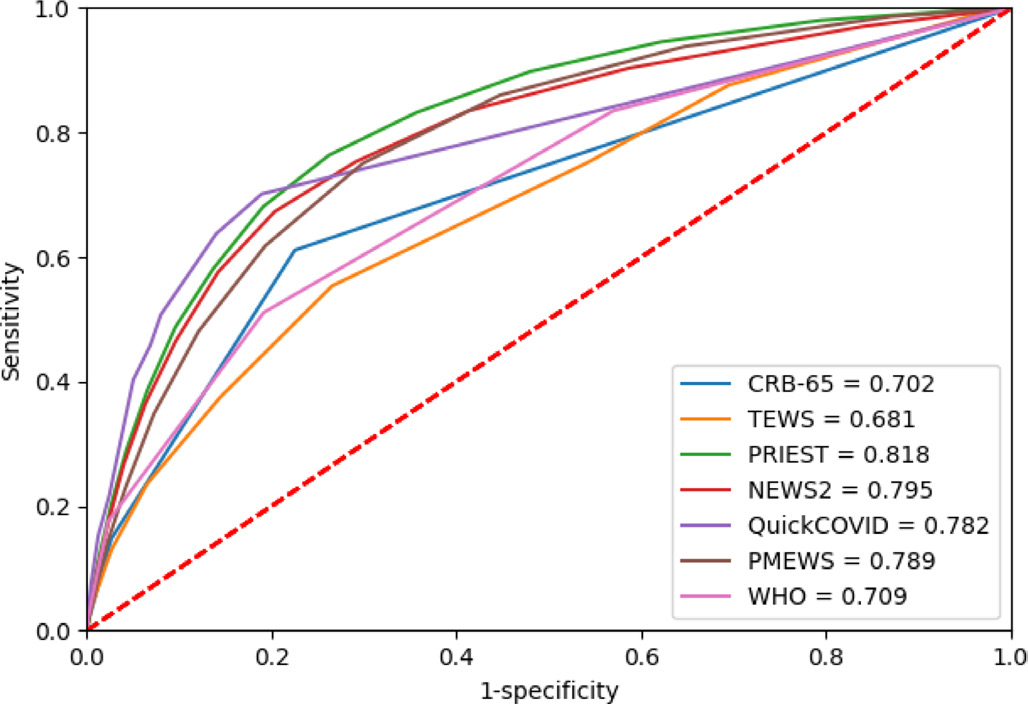
Performance of tools predicting composite primary outcome for total study period. NEWS2, National Early Warning Score, version 2; PMEWS, Pandemic Medical Early Warning Score; PRIEST, Pandemic Respiratory Infection Emergency System Triage; TEWS, Triage Early Warning Score; CRB-65, Confusion Respiratory Rate Blood Pressure-65.

**Figure 3:**
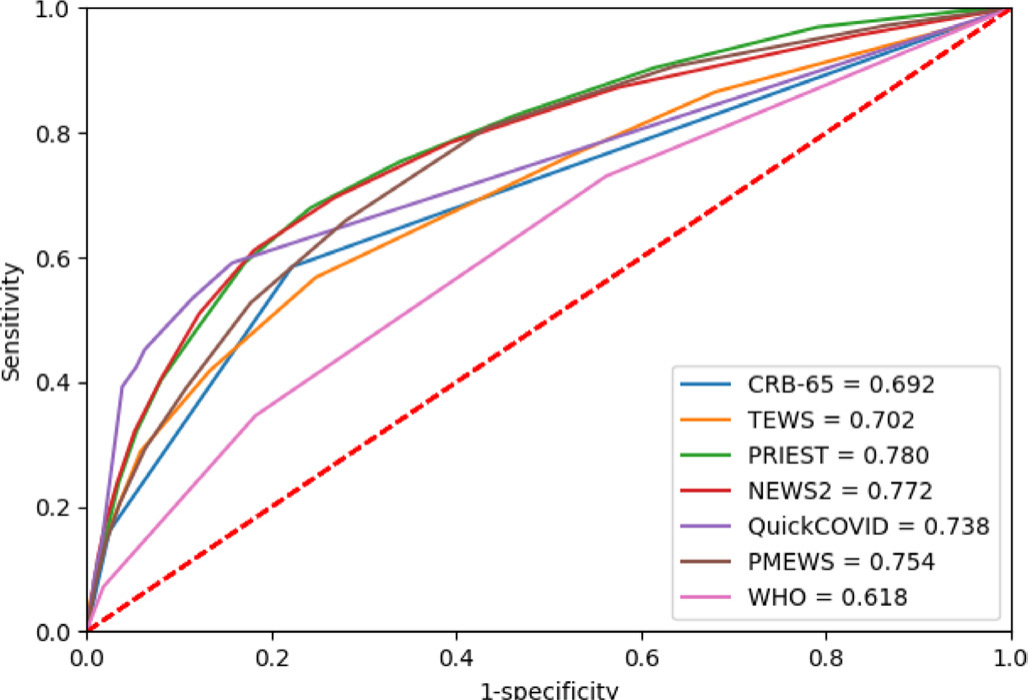
Performance of tools predicting composite primary outcome for the Omicron period. NEWS2, National Early Warning Score, version 2; PMEWS, Pandemic Medical Early Warning Score; PRIEST, Pandemic Respiratory Infection Emergency System Triage; TEWS, Triage Early Warning Score; CRB-65, Confusion Respiratory Rate Blood Pressure Age≥65.

Sensitivity, specificity, PPV and NPV for predicting the primary composite outcome using the previously recommended score thresholds are provided in table 2 and for the Omicron period in table 3. Sensitivity and specificity statistics are provided for every score threshold in online supplemental materials 2 and 3. The ROC curves for these analyses are shown in figures 2 and 3. Calibration curves for the primary outcome are provided in online supplemental material 4.

**Table 2:** Triage tool diagnostic accuracy statistics (95% CI) for predicting any adverse outcome (entire study period)

Tool	N*	C-statistic	Threshold	N (%) above threshold	Sensitivity	Specificity	PPV	NPV
CRB-65	432 580	0.70 (0.70, 0.71)	>0	102 964 (23.8%)	0.61 (0.60, 0.62)	0.78 (0.77, 0.78)	0.09 (0.09, 0.09)	0.98 (0.98, 0.98)
NEWS2	433 079	0.80 (0.79, 0.80)	>1	258 643 (59.7%)	0.90 (0.90, 0.91)	0.41 (0.41, 0.42)	0.05 (0.05, 0.05)	0.99 (0.99, 0.99)
PMEWS	438 806	0.79 (0.79, 0.79)	>2	202 335 (46.11 %)	0.86 (0.85, 0.87)	0.55 (0.55, 0.55)	0.06 (0.06, 0.07)	0.99 (0.99,0.99)
PRIEST	438 876	0.82 (0.82, 0.82)	>4	163 649 (37.3%)	0.83 (0.83, 0.84)	0.64 (0.64,0.64)	0.08 (0.08, 0.08)	0.99 (0.99, 0.99)
WHO	437 846	0.71 (0.71, 0.72)	>0	253 355 (57.9%)	0.84 (0.83, 0.84)	0.43 (0.43, 0.43)	0.05 (0.05, 0.05)	0.99 (0.99, 0.99)
TEWS	432 606	0.68 (0.68, 0.69)	>2	237 482 (31%)	0.75 (0.74, 0.76)	0.46 (0.46, 0.46)	0.05 (0.05, 0.05)	0.98 (0.98, 0.98)
Quick COVID	446 084	0.78 (0.78, 0.79)	>3	36 634 (8.2)	0.45 (0.45, 0.47)	0.93 (0.93, 0.93)	0.19 (0.19, 0.20)	0.98 (0.98, 0.98)

*Patients with <3 parameters were excluded from analysis when estimating performance.

CRB-65, Confusion Respiratory Rate Blood Pressure Age≥65; NEWS2, National Early Warning Score, version 2; NPV, negative predictive value; PMEWS, Pandemic Medical Early Warning Score; PPV, positive predictive value; PRIEST, Pandemic Respiratory Infection Emergency System Triage; TEWS, Triage Early Warning Score.

**Table 3:** Triage tool diagnostic accuracy statistics (95% CI) for predicting any adverse outcome (Omicron period)

Tool	N*	C-statistic	Threshold	N (%) above threshold	Sensitivity	Specificity	PPV	NPV
CRB-65	136 961	0.69 (0.68, 0.70)	>0	31 373 (22.9%)	0.59 (0.57, 0.60)	0.78 (0.78, 0.78)	0.05 (0.05, 0.05)	0.99 (0.99, 0.99)
NEWS2	137 116	0.77 (0.76, 0.78)	>1	79 487 (58%)	0.87 (0.86, 0.88)	0.43 (0.42, 0.43)	0.03 (0.03, 0.03)	0.99 (0.99, 0.99)
PMEWS	138 954	0.75 (0.74, 0.76)	>2	60 990 (43.9%)	0.81 (0.79, 0.82)	0.57 (0.57, 0.57)	0.04 (0.04, 0.05)	0.99 (0.99, 0.99)
PRIEST	138 982	0.78 (0.77, 0.79)	>4	48 134 (34.6%)	0.75 (0.74, 0.77)	0.66 (0.66, 0.66)	0.04 (0.04, 0.04)	0.99 (0.99, 0.99)
WHO	138 666	0.62 (0.61, 0.63)	>0	78 355 (56.5%)	0.73 (0.71, 0.75)	0.44 (0.44, 0.44)	0.03 (0.02, 0.03)	0.99 (0.99, 0.99)
TEWS	136 967	0.70 (0.69, 0.71)	>2	72 750 (53.1%)	0.77 (0.75, 0.78)	0.47 (0.47, 0.48)	0.03 (0.03, 0.03)	0.99 (0.99, 0.99)
Quick COVID	140 520	0.74 (0.73, 0.75)	>3	8529 (6.1%)	0.42 (0.41, 0.44)	0.95 (0.95, 0.95)	0.14 (0.13, 0.15)	0.99 (0.99, 0.99)

*Patients with <3 parameters were excluded from analysis when estimating performance.

CRB-65, Confusion Respiratory Rate Blood Pressure Age≥65; NEWS2, National Early Warning Score, version 2; NPV, negative predictive value; PMEWS, Pandemic Medical Early Warning Score; PPV, positive predictive value; PRIEST, Pandemic Respiratory Infection Emergency System Triage; TEWS, Triage Early Warning Score.

Sensitivity, specificity, PPV and NPV for predicting the secondary outcomes (death and ICU admission) using recommended score thresholds are presented in table 4 and table 5. The accompanying ROC curves are presented in online supplemental material 5 and 6.

**Table 4:** Triage tool diagnostic accuracy statistics (95% CI) for predicting death (entire study period)

Tool	N*	C-statistic	Threshold	N (%) above threshold	Sensitivity	Specificity	PPV	NPV
CRB-65	432 580	0.70 (0.69, 0.70)	>0	102 964 (23.8%)	0.60 (0.59, 0.61)	0.77 (0.77, 0.77)	0.06 (0.06, 0.06)	0.99 (0.99, 0.99)
NEWS2	433 079	0.78 (0.78, 0.79)	>1	258 643 (59.7)	0.89 (0.89, 0.90)	0.41 (0.41, 0.41)	0.04 (0.04, 0.04)	0.99 (0.99, 0.99)
PMEWS	438 806	0.81 (0.80, 0.81)	>2	202 335 (46.11%)	0.88 (0.87, 0.89)	0.55 (0.55, 0.55)	0.05 (0.05, 0.05)	0.99 (0.99, 0.99)
PRIEST	438 876	0.83 (0.83, 0.83)	>4	163 649 (37.3%)	0.85 (0.85, 0.86)	0.64 (0.64, 0.64)	0.06 (0.06, 0.06)	0.99 (0.99, 0.99)
WHO	437 846	0.79 (0.79, 0.80)	>0	253 355 (57.9%)	0.94 (0.94, 0.95)	0.43 (0.43, 0.43)	0.04 (0.04, 0.04)	0.996 (0.996, 0.997)
TEWS	432 606	0.65 (0.65, 0.66)	>2	237 482 (54.9%)	0.72 (0.72, 0.73)	0.46 (0.45, 0.46)	0.03 (0.03, 0.03)	0.99 (0.99, 0.99)
Quick COVID	446 084	0.76 (0.75, 0.76)	>3	36 634 (8.2%)	0.36 (0.35, 0.37)	0.93 (0.92, 0.93)	0.11 (0.11, 0.11)	0.98 (0.98, 0.98)

*Patients with <3 parameters were excluded from analysis when estimating performance.

CRB-65, Confusion Respiratory Rate Blood Pressure Age≥65; NEWS2, National Early Warning Score, version 2; NPV, negative predictive value; PMEWS, Pandemic Medical Early Warning Score; PPV, positive predictive value; PRIEST, Pandemic Respiratory Infection Emergency System Triage; TEWS, Triage Early Warning Score.

**Table 5:** Triage tool diagnostic accuracy statistics (95% CI) for predicting ICU admission (entire study period)

Tool	N*	C-statistic	Threshold	N (%) above threshold	Sensitivity	Specificity	PPV	NPV
CRB-65	432 580	0.57 (0.57, 0.58)	>0	102 964 (23.8%)	0.38 (0.36, 0.40)	0.76 (0.76, 0.76)	0.008 (0.007, 0.009)	0.996 (0.996 0.996)
NEWS2	433 079	0.72 (0.70, 0.73)	>1	258 643 (59.7)	0.85 (0.83, 0.86)	0.40 (0.40, 0.41)	0.007 (0.007, 0.007)	0.993 (0.993, 0.993)
PMEWS	438 806	0.71 (0.70, 0.72)	>2	202 335 (46.1%)	0.76 (0.75, 0.78)	0.54 (0.54, 0.54)	0.008 (0.008, 0.009)	0.998 (0.997, 0.998)
PRIEST	438 876	0.71 (0.70, 0.71)	>4	163 649 (37.3%)	0.67 (0.65, 0.69	0.63 (0.63, 0.63)	0.009 (0.008, 0.009)	0.997 (0.997, 0.998)
WHO	437 850	0.63 (0.61, 0.64)	>0	253 355 (57.9%)	0.77 (0.75, 0.79)	0.42 (0.42, 0.42)	0.007 (0.006, 0.007)	0.997 (0.997, 0.998)
TEWS	432 606	0.66 (0.65, 0.67)	>2	237 482 (54.9%)	0.75 (0.73, 0.77)	0.45 (0.45, 0.45)	0.007 (0.006, 0.007)	0.997 (0.997, 0.997)
Quick COVID	446 084	0.66 (0.65, 0.66)	>3	36 634 (8.2%)	0.30 (0.28, 0.32)	0.92 (0.92, 0.92)	0.02 (0.02, 0.02)	0.996 (0.996, 0.996)

*Patients with <3 parameters were excluded from analysis when estimating performance.

CRB-65, Confusion Respiratory Rate Blood Pressure Age≥65; ICU, intensive care unit; NEWS2, National Early Warning Score, version 2; PMEWS, Pandemic Medical Early Warning Score; PRIEST, Pandemic Respiratory Infection Emergency System Triage; TEWS, Triage Early Warning Score.

Clinical decision-making to admit patients to hospital from the ED had a sensitivity of 0.77 (95% CI 0.76 to 0.78) and specificity 0.88 (95% CI 0.87 to 0.88) for the primary outcome. The PPV was 0.18 (95% CI 0.18 to 0.18) and the NPV was 0.99 (95% CI 0.99 to 0.99). Hypothetical use of the PRIEST tool, NEWS2 and PMEWS triage tools would have achieved a higher sensitivity than existing clinical practice to the primary outcome across the study period but this was at a cost of a lower specificity (table 2). Use of these tools would have more than doubled admissions with only a small reduction in risk of false negative triage. The triage tools generally demonstrated worse discrimination (except TEWS2) during the Omicron period and for ICU admission. The tools (apart from the Quick-COVID score) all had better discrimination, higher sensitivity but lower specificity when predicting death compared with other outcomes (table 4). Calibration slopes showed that all tools, apart from the WHO score, overpredicted risk at higher scores (online supplemental material 4).

## Discussion

### Summary

This large retrospective cohort study conducted in a middle-income setting includes data collected from August 2020 to March 2022, encompassing patients with confirmed or clinically suspected COVID from the Beta, Delta and Omicron waves in the Western Cape.^21^ The estimated rate of the primary outcome (death, respiratory support or ICU admission) was 3.45% (95% CI 3.4% to 3.51%) across the study period and 1.98% (95% CI 1.91% to 2.06%) for patients who presented during the Omicron period (31.5% of the cohort).

Existing clinical decision-making only achieved a sensitivity of 0.77 (95% CI 0.76 to 0.78) to the primary outcome, meaning 3535 patients (22.93%) with adverse outcomes were not initially admitted to hospital. The low prevalence of the primary outcome meant that 85.28% of patients were discharged on first presentation and discharged patients had less than a 1% chance of experiencing the primary outcome (NPV 0.99, 95% CI 0.99 to 0.99). Use of the PRIEST, NEWS2 and PMEWS tools at recommended score thresholds would have improved sensitivity; however, this would have caused an increase in hospital admissions of between 22.6% (PRIEST) and 45% (NEWS2), with only modest associated gains in NPV (table 2). Potentially using these scores at higher than recommended thresholds would not increase admissions with an associated risk of false negative triage similar to current clinical practice (table 2 and online supplemental material 7).

### Comparison to previous literature

The adverse outcome rate estimated for this study (3.45%) is lower than comparable studies conducted in Europe during the first wave of pandemic.^25 26^ A UK study reported that 22.1% patients with suspected COVID died or required organ support in an ED setting.^26^ The lower adverse event rate is partly explained by the majority (76.6%) of our study cohort being selected after the second COVID wave which ended in March 2021. Inpatient case fatality rates in South Africa for patients with confirmed COVID had fallen from a high of 28.8% (second wave) to 21.5% during the third wave (April to November 2021) and were estimated to be 10.7% during the Omicron period.^27^ Only 14.7% of our cohort were admitted for inpatient care. In European and other high-income settings, the use of telephone triage and other measures may have acted to prevent lower risk patients with suspected COVID from attending the ED.^28^ In South Africa, equivalent community advice and triage services were not available and this resulted in large numbers of lower acuity patients self-presenting to hospital.^19^ In our study, 91.9% of patients did not require supplemental oxygen on arrival to ED compared with 68.4% in the UK PRIEST study.^4^

The PRIEST tool is recommended by the American College of Emergency Physicians to aid risk-stratification of patients with suspected COVID.^6^ The score has been externally validated in a UK prehospital alpha wave cohort and a cohort of 306 patients presenting to EDs in the USA in the winter of 2020-21.^22 29^ In the development study, the PRIEST score achieved a C-statistic of 0.80 (95% CI 0.79 to 0.81) and, at the recommended threshold of a score above 4 points, a sensitivity 0.98 (95% CI 0.97 to 0.98) and specificity 0.34 (95% CI 0.34 to 0.35) for a composite outcome of death or organ support.^4^ In the American validation study, the score achieved a C-statistic of 0.86 (95% CI 0.81 to 0.91) and sensitivity of 97.7% (95% CI 93.2% to 100%) and specificity of 47.2% (95% CI 41.1% to 53.2%) for a similar outcome.^29^ In a UK prehospital study, the PRIEST score achieved a C-statistic of 0.83 (0.82 to 0.84), sensitivity of 0.97 (95% CI 0.97 to 0.97) and specificity of 0.41 (95% CI 0.40 to 0.41).^22^

In this study cohort, the PRIEST score (without inclusion of performance status) achieved the best overall discrimination (C-statistic 0.82 (95% CI 0.82 to 0.82)) and sensitivity 0.83 (95% CI 0.83 to 0.84) and specificity of 0.64 (95% CI 0.64 to 0.66) for the primary outcome. Although the discrimination is similar to previous studies, differences in the NPV and PPV in our study are not explainable solely by the lower prevalence of adverse outcomes. Our study population had several differences to the PRIEST derivation cohort, including younger average age (mean 43.6 years (SD 17.2) vs 62.4 (SD 19.7) in the UK PRIEST cohort) and less impaired physiology (42% of patients NEWS 0 or 1 compared 23% patients in UK PRIEST cohort).

### Strengths and limitations

This is the first study to use a large cohort of patients identified using routinely collected electronic healthcare data to validate triage tools in patients with suspected COVID in the Western Cape in the ED setting. Our cohort is based on the clinical impression of likely COVID infection as determined by the clinical staff performing the initial triage of patients in the ED. Clinical suspicion was partly determined by the prevalence of COVID-19 infection which varied during the study period and hospital guidelines which were updated to account for changes in symptoms associated with COVID. The use of a cohort of patients with suspected infection is important, as this reflects the population which ED staff must clinically triage.^1^ Other prediction models, such as the International Severe Acute Respiratory Infection Consortium (Coronavirus Clinical Characterisation Consortium) (ISARIC 4C) prediction model, require investigations, including blood tests, and are intended for prediction of inpatient mortality in patients with confirmed COVID, and not rapid triage of need for admission in an ED setting.^30^ We had low rates of missing data in risk score variables (table 1). Our dataset also comprises multiple COVID waves and allowed the comparison of triage tool performance in the Omicron and earlier waves.^21^ Although we have compared the accuracy of different triage scores to each other, and to existing clinical practice, prospective prognostic impact studies are required to determine if use of such scores leads to improved patient outcomes or cost-effectiveness.^31^ Equally, although we have followed most recommendations regarding prognostic model validation and present the performance of triage tools across a range of available scores, we have not performed decision curve analysis.^31 32^

Our study only includes government hospitals which use the HECTIS system in the Western Cape, and our outcomes are limited to those recorded in hospital. Consequently, deaths are only recorded if the death occurred at, or was specifically notified to, a health facility. Deaths at home are not included. Given the substantial increase in excess deaths attributed to COVID in South Africa (likely at least 68% increase for the Western Cape) which occurred undiagnosed at home, it is likely that there were more deaths within 30 days than reflected by the data and the performance of current clinical judgement may be overestimated.^33^ However, undiagnosed community deaths due to COVID for patients who never attended hospital will not affect estimates of clinical or risk score accuracy. Implementation of the HECTIS system means that the majority of our study population was included after March 2021. Use of the HECTIS system, electronic records and linking of data from various sources is in its infancy in this context and we are unable to verify the accuracy of individual data, and dependent on a large number of data entry points across facilities and institutions. However, the HECTIS system is used clinically to collect and record the physiological and other variables used to calculate SATS at initial triage in the ED.^7^ Other variables may be recorded less accurately. We assumed that if comorbidities were not recorded in the routine datasets, they were not present.

### Implications

The PRIEST, NEWS2 and PMEWS triage tools all achieved C-statistics of around 0.8 when estimating our primary outcome and may show sufficient accuracy to be used clinically in the Western Cape. However, in settings with a similarly low prevalence of death or organ support as this study (3.45% vs 22.1% PRIEST tool development study), use of these tools at previously recommended thresholds would cause large increases in hospital admissions with very small associated gains in reduced risk of false negative triage. The lower risk of death and ICU admission in this study may reflect the role telephone and other prehospital triage had in reducing ED attendances of lower-risk patients in the UK and other settings and the lower severity associated with the Omicron wave.^22 28^ This highlights the need to validate triage tools in different settings, populations and waves to ensure accuracy of risk prediction as the results of of external validation may not be generalisable^2^

Current clinical decision-making to admit patients in this setting achieved a sensitivity of 0.77 (95% CI 0.76 to 0.78) and specificity 0.88 (95% CI 0.87 to 0.88) for the primary outcome. The low prevalence of the primary outcome meant this achieved a NPV of 0.99 (95% CI 0.99 to 0.99) and only 14.7% of patients were admitted. No risk score outperformed clinical decision-making. Use of the PRIEST score at a point higher (0–5 vs 6+) would achieve performance most similar to current practice (27.9% of patients admitted with a NPV of 98.9%) (table 2 and online supplemental material 2).

Previous literature reviews have found that clinical decision rules and risk scores rarely outperform clinician gestalt diagnostically or in predicting outcomes.^34^ Our study supports this finding. However, use of the PRIEST tool in a prehospital UK setting was found to potentially reduce the risk of false negative triage without causing a large increase in ambulance conveyance.^22^ Risk scores may improve speed, reproducibility and transparency of decision-making, especially for less experienced clinicians.^2^ The PRIEST score and PMEWS also use predictors, such as performance status, which are not routinely collected in this setting. Development of a triage tool in this setting based on existing triage practice and routinely collected predictive clinical information may improve accuracy and applicability.

The primary outcome used in this study is composite of intubation or non-invasive ventilation in the ED, death and ICU admission (a surrogate for organ support), as this was thought to encompass a definite need for hospital admission.^4^ However, all tools predicted death better than ICU admission (tables 4 and 5). This may reflect some predictors, such as age, included in risk scores, being much stronger predictors of death (including unavoidable deaths) than ventilatory support or ICU admission.^35^ It is therefore important that accuracy of the tools for the primary composite outcome is not used to guide treatment decisions beyond the need for admission, such as need for organ support or treatment in an ICU setting, as potential benefit may be overestimated.

## Conclusion

The NEWS2, PMEWS and PRIEST tools achieved good estimated discrimination with respect to death or organ support. However, in part due to the low prevalence of the primary outcome, use of these tools at previously recommended thresholds would lead to a large increase in hospital admission with a very small associated reduction in false negative triage. No risk score outperformed existing clinical discharge decision-making in this setting.

## Data Availability

Data may be obtained from a third party and are not publicly available. The data used for this study are subject to a data sharing agreement with the Western Cape Government Department of Health and Wellness, which prohibits further sharing of patient-level data. Access to these and related data should be requested directly from this organisation and is subject to the necessary ethical and organisational approval processes.
