# Supplementary material for: External validation of triage tools for adults with suspected COVID-19 in a middle-income setting: an observational cohort study
Source: Emerg Med J. 2023 May 22;40(7):509–17. doi: 10.1136/emermed-2022-212827 (PMC10359554; doi:10.1136/emermed-2022-212827)
Supplement: Supplementary data [file emermed-2022-212827supp001.pdf]

Supplementary Material 1: Triage tool scoring details

CRB-65:

The CRB-65 score is recommended for community settings where access to blood testing is limited. The CRB-65 score uses four parameters, each scoring 1 point when positive and zero if negative, to give a total score between zero and four. It is a 4-point scale that does not include urea with the threshold as <2 for low risk, 2+ for high risk

Four parameters:

1. Confusion: GCS-V is less than 4 or GCS total is less than 15 or AVPU is recorded as V, P or U
2. Respiratory: rate of 30 breaths per minute or more
3. Blood pressure: diastolic BP is 60mmHg or less or systolic BP is 90 mmHg or less
4. Age: 65 years or more

Missing data: If a patient has fewer than three of the four parameters complete, the score was not calculated, otherwise values were assumed to be zero.

PMEWS:

PMEWS uses six physiological parameters and patient parameters to calculate a score from zero to 19. The score is calculated by taking the score in the table below dependent on each of the six physiological parameters then adding points for two patient parameters after if they are positive. As living circumstances and performance status were not available in the routine dataset used they were not included in the calculation of the score.

Physiological:

Score	3	2	1	0	1	2	3
**Respiratory Rate**	≤8			9-18	19-25	26-29	≥30
**SaO_2**	≤89	90-93	94-96	>96			
**Pulse Rate**	≤40	41-50		51-100	101-110	111-129	≥130
**Systolic BP**	≤70	71-90	91-100	>100			
**Temperature**		≤35.0	35.1-36.0	36.1-37.9	38-38.9	≥39	
**Neuro**				Alert	Confused Agitated*	Voice	Pain Uncon

* confused/agitated will be defined based on GCS-V<4 or GCS total<15

Patient:

1. Add 1 point if age>65
2. Add 1 point if either:
   a. Patient lives alone / no fixed abode or
   b. has a co-morbidity (respiratory, cardiac, renal, immunosuppressed, diabetes)
   c. performance status is more than two suggesting limited activity can self-care, limited activity limited self-care, or bed/chair bound no self-care.

Missing data: If data is missing one or two variables then the normal score (zero) was assumed. If less than three parameters were available the patient was excluded.

NEWS2:

The NEWS2 has seven parameters which are scores from zero to three providing an overall score between zero and 20. The scores for each parameter can be found in the table below.

Score	3	2	1	0	1	2	3
**Respiratory Rate**	≤8		9-11	12-20		21-24	≥25
**SaO_2**	≤91	92-93	94-95	≥96			
**Pulse Rate**	≤40		41-50	51-90	91-110	111-130	≥131
**Systolic BP**	≤90	91-100	101-110	111-219			≥220
**Temperature**	≤35.0		35.1-36.0	36.1-38.0	38.1-39.0	≥39.1	
**Neuro**				Alert			Confusion, Voice, Pain, Unresponsive
**Air or Oxygen**		Oxygen (based on FiO_2>21%, or FiO_2>0 L/min)		Air			

Missing data: Any missing data will be imputed with the value zero, therefore classifying missing as normal. The score was not calculated if fewer than three of the parameters were available.

WHO decision making algorithm for hospitalisation with pneumonia:

The WHO decision making algorithm for hospitalisation with pneumonia suggests an adult patient is admitted (score 1) if any of the following are present:

- respiratory rate >30/minute,
- oxygen saturation <90%,
- respiratory distress (not included in this evaluation),
- age >60,
- any of the following comorbidities; hypertension, diabetes, cardiovascular disease, chronic respiratory disease, renal impairment or immunosuppression

As a subjective clinical assessment of respiratory distress is not routinely recorded in our data this was not included. Any missing data was assumed as normal. A score was not calculated if fewer than three of the parameters was available.

The PRIEST clinical severity Score:

The core PRIEST clinical severity score consists of the seven parameters of NEWS2 and age, sex and performance status. As performances status was not available in the routine data used, this was not included. Scores for each parameter are as follows.

**Variable**	**Range**	**Score**
Respiratory rate (per minute)	12-20	0
	9-11	1
	21-24	2
	<9 or >24	3
Oxygen saturation (%)	>95	0
	94-95	1
	92-93	2
	<92	3
Heart rate (per minute)	51-90	0
	41-50 or 91-110	1
	111-130	2
	<41 or >130	3
Systolic BP (mmHg)	111-219	0
	101-110	1
	91-100	2
	<91 or >219	3

Temperature (C)	36.1-38.0	0
	35.1-36.0 or 38.1-39.0	1
	>39.0	2
	<35.1	3
Alertness	Alert	0
	Confused or not alert	3
Inspired oxygen	Air	0
	Supplemental oxygen	2
Sex	Female	0
	Male	1
Age (years)	16-49	0
	50-65	2
	66-80	3
	>80	4
Performance status	Unrestricted normal activity	0
	Limited strenuous activity, can do light activity	1
	Limited activity, can self-care	2
	Limited self-care	3
	Bed/chair bound, no self-care	4

Any missing data was assumed as normal. A score was not calculated if fewer than three of the parameters was available.

Quick COVID-19 Severity Index (qCSI)

The qCSI was developed to predict predict respiratory failure within 24 hours of ED attendance as defined by oxygen requirement of greater than 10 L/min by low-flow device, high-flow device, non-invasive or invasive ventilation, or death. The qCSI has three parameters which are scores from zero to five providing an overall score between zero and nine. The scores for each parameter can be found in the table below.

**Score**	**2**	**1**	**0**	**1**	**2**	**3**	**4**	**5**
**Respiratory Rate**			≥22	23-28	>28			
**SaO_2**	≤88	89-92	>92					

**Oxygen Flow Rate Litres/min**			≤2				3-4	>4

As there are only three components, missing values were assumed to be in normal range and score zero.

Triage Early Warning Score (TEWS)

TEWS is a validated composite triage score which form part of the South African Triage Score, based on judgement of the patient's vital parameters (respiratory rate, heart rate, temperature, systolic blood pressure) and level of consciousness. Possible sum scores range from 0 to 14.

**Score**	**3**	**2**	**1**	**0**	**1**	**2**	**3**
**Respiratory Rate**		<9		9-14	15-20	21-29	>29
**Pulse Rate**		<41	41-50	51-100	101-110	111-129	>129
**Systolic BP**	≤70	71-80	81-100	101-199		>199	
**Temperature**		<35		35-38.4		≥38.5	
**Neuro**		Confused		Alert	Reacts to Voice	Reacts to pain	Unresponsive

Any missing data was assumed as normal. A score was not calculated if fewer than three of the parameters was available

Supplementary Material 2: Performance of triage tools across the whole range of available scores (overall time period)

			Any adverse outcome			
**Tool**	**Threshold**	**N (%) above threshold**	**Sensitivity (%)**	**Specificity (%)**	**Positive Predictive Value (%)**	**Negative Predictive Value (%)**
CRB-65	>0	102964 (23.8%)	61.1	77.5	8.6	98.3
	>1	13135 (3.0%)	14.7	97.4	16.3	97
	>2	1053 (0.2%)	1.7	99.8	22.8	96.7
	>3	37 (0.0%)	0.07	99.99	27	96.6
NEWS2	>0	365445 (84.4%)	97.1	16.1	3.9	99.4
	>1	258643 (59.7%)	90.4	41.4	5.1	99.2
	>2	185210 (42.8%)	83.5	58.7	6.6	99.
	>3	132972 (30.7%)	75.4	70.9	8.3	98.8
	>4	94906(21.9%)	67.3	79.7	10.4	98.6
	>5	67910(15.7%)	57.6	85.8	12.4	98.3
	>6	47145(10.9%)	46.6	90.4	14.5	98
	>7	31845(7.4%)	36.5	93.7	16.8	97.7
	>8	20595(4.8%)	26.7	96	19	97.4
	>9	12559(2.9%)	17.7	97.6	20.7	97.1
	>10	7548(1.7%)	11.5	98.6	22.2	97
	>11	4411(1.0%)	7.2	99.2	23.9	96.8
	>12	2615(0.6%)	4.6	99.6	25.9	96.8
	>13	1421(0.3%)	2.6	99.8	27.1	96.7
	>14	766(0.2%)	1.6	99.9	30.7	96.7
	>15	394(0.1%)	1	99.9	37.1	96.7
	>16	181(0.0%)	0.4	99.97	33.7	96.6
	>17	66(0.0%)	0.2	99.99	34.9	96.6
	>18	24(0.0%)	0.04	100	25	96.6
PMEWS	>0	383454 (87.4%)	98.6	13	3.8	99.6
	>1	288311 (65.7%)	93.9	35.3	4.9	99.4
	>2	202335 (46.1%)	86	55.3	6.4	99.1
	>3	137726 (31.4%)	75.1	70.2	8.1	98.8
	>4	91064(20.8%)	61.8	80.7	10.1	98.4
	>5	58339(13.3%)	48	87.9	12.3	98
	>6	35875(8.2%)	34.8	92.8	14.5	97.6
	>7	21078(4.8%)	22.9	95.8	16.2	97.2
	>8	11816(2.7%)	14.4	97.7	18.2	97

			Any adverse outcome			
**Tool**	**Threshold**	**N (%) above threshold**	**Sensitivity (%)**	**Specificity (%)**	**Positive Predictive Value (%)**	**Negative Predictive Value (%)**
	>9	6196(1.4%)	8	98.8	19.3	96.8
	>10	3084(0.7%)	4.2	99.4	20.4	96.7
	>11	1447(0.3%)	2.2	99.7	22.5	96.7
	>12	652(0.1%)	1	99.9	23.6	96.6
	>13	263(0.1%)	0.4	99.95	22.4	96.6
	>14	131(0.0%)	0.3	99.98	29	96.6
	>15	31(0.0%)	0.1	100	41.9	96.6
	>16	4(0.0%)	0.01	100	50	96.6
PRIEST	>0	416143 (94.8%)	99.6	5.4	3.6	99.8
	>1	351143 (80%)	98	20.6	4.2	99.7
	>2	277619 (63.3%)	94.6	37.9	5.1	99.5
	>3	216881 (49.4%)	89.8	52	6.2	99.3
	>4	163649 (37.3%)	83.2	64.3	7.6	99.1
	>5	122445 (27.9%)	76.4	73.8	9.3	98.9
	>6	91077(20.8%)	68.1	80.9	11.2	98.6
	>7	66566(15.2%)	58.1	86.4	13.1	98.3
	>8	47861(10.9%)	48.8	90.4	15.2	98.04
	>9	33488(7.6%)	38.6	93.5	17.2	97.7
	>10	22297(5.1%)	28.9	95.8	19.4	97.5
	>11	14166(3.2%)	20.2	97.4	21.3	97.2
	>12	8514(1.9%)	13.3	98.5	23.4	97
	>13	5250(1.2%)	8.9	99.1	25.4	96.9
	>14	3142(0.7%)	5.6	99.5	26.8	96.8
	>15	1902(0.4%)	3.5	99.7	27.5	96.7
	>16	1072(0.2%)	2	99.8	27.6	96.7
	>17	570(0.1%)	1.1	99.9	29	96.6
	>18	295(0.1%)	0.6	99.95	29.8	96.6
WHO	>0	253355 (57.9%)	83.52	43.03	4.87	98.68
TEWS	>0	413795 (95.7%)	98.7	4.5	3.5	99
	>1	303017 (70.0%)	87.6	30.6	4.2	98.6
	>2	237482 (54.9%)	75.2	45.8	4.6	98.2
	>3	118976 (27.5%)	55.4	73.5	6.8	97.9
	>4	65834	37.5	85.6	8.3	97.5

			Any adverse outcome			
**Tool**	**Threshold**	**N (%) above threshold**	**Sensitivity (%)**	**Specificity (%)**	**Positive Predictive Value (%)**	**Negative Predictive Value (%)**
		(15.2%)				
	>5	30562(7.1%)	23.5	93.5	11.2	97.2
	>6	13156(3.0%)	13.1	97.3	14.5	97
	>7	5372(1.2%)	6.3	98.9	17	96.8
	>8	2076(0.5%)	3.2	99.6	22.2	96.7
	>9	781(0.2%)	1.4	99.9	25.9	96.7
	>10	277(0.1%)	0.6	99.95	30	96.7
	>11	92(0.0%)	0.2	99.98	28.3	96.6
	>12	18(0.0%)	0.04	100	33.3	96.6
Quick COVID	>0	92282(20.7%)	70.1	81.1	11.7	98.7
	>1	70045(15.7%)	63.7	86.	14	98.5
	>2	42274(9.5%)	50.7	92	18.5	98.1
	>3	36634(8.2%)	45.9	93.1	19.3	98
	>4	27798(6.2%)	40.3	95	22.3	97.8
	>5	13871(3.1%)	21.8	97.6	24.2	97.2
	>6	7635(1.7%)	15.3	98.8	30.8	97
	>7	3171(0.7%)	7.7	99.5	37.3	96.8
	>8	1256(0.3%)	3.4	99.8	42	96.7

Supplementary Material 3: Performance of triage tools across the whole range of available scores (Omicron period)

			Any adverse outcome			
**Tool**	**Threshold**	**N (%) above threshold**	**Sensitivity (%)**	**Specificity (%)**	**Positive Predictive Value (%)**	**Negative Predictive Value (%)**
CRB-65	>0	31373(22.9%)	58.5	77.8	5	98.9
	>1	3699(2.7%)	16.1	97.6	11.7	98.3
	>2	299(0.2%)	1.3	99.8	12	98.1
	>3	12(0.0%)	0.2	99.99	33.3	98
NEWS2	>0	114879 (83.8%)	95.7	16.5	2.3	99.5
	>1	79487(58%)	87.2	42.6	3	99.4
	>2	55011(40.1%)	78.5	60.7	3.9	99.3
	>3	37981(27.7%)	69.7	73.2	5	99.2
	>4	26010(19%)	61.2	81.9	6.4	99.1
	>5	17761(13%)	51.1	87.8	7.8	98.9
	>6	11894(8.7%)	40.5	92	9.3	98.7
	>7	7792(5.7%)	32.1	94.9	11.2	98.6

			Any adverse outcome			
**Tool**	**Threshold**	**N (%) above threshold**	**Sensitivity (%)**	**Specificity (%)**	**Positive Predictive Value (%)**	**Negative Predictive Value (%)**
	>8	5051(3.7%)	23.9	96.7	12.9	98.4
	>9	3050(2.2%)	16.9	98.1	15.1	98.3
	>10	1905(1.4%)	11.7	98.8	16.8	98.2
	>11	1130(0.8%)	7.2	99.3	17.3	98.1
	>12	697(0.5%)	4.3	99.6	16.9	98.1
	>13	370(0.3%)	2.4	99.8	17.6	98.1
	>14	201(0.1%)	1.4	99.9	18.4	98.04
	>15	84(0.1%)	0.9	99.96	29.8	98.03
	>16	52(0.0%)	0.6	99.97	32.7	98.03
	>17	32(0.0%)	0.4	99.98	34.4	98.02
	>18	8(0.0%)	0.1	100	25	98.02
PMEWS	>0	120373 (86.6%)	97.2	13.6	2.2	99.6
	>1	88902(64.0%)	90.6	36.6	2.8	99.5
	>2	60990(43.9%)	80.7	56.9	3.7	99.3
	>3	39937(28.7%)	65.9	72	4.6	99.1
	>4	25595(18.4%)	52.7	82.3	5.7	98.8
	>5	15675(11.3%)	39	89.3	6.9	98.6
	>6	9398(6.8%)	29.3	93.7	8.7	98.5
	>7	5418(3.9%)	20.6	96.4	10.5	98.4
	>8	2969(2.1%)	13.6	98.1	12.7	98.2
	>9	1573(1.1%)	7.8	99	13.7	98.1
	>10	843(0.6%)	4.6	99.5	15	98.1
	>11	378(0.3%)	2	99.8	14.3	98.04
	>12	183(0.1%)	0.9	99.9	14.2	98.02
	>13	82(0.1%)	0.5	99.95	17.1	98.01
	>14	47(0%)	0.3	99.97	17	98.01
	>15	12(0%)	0.1	99.99	33.3	98.01
	>16	2(0%)	0.07	100	100	98.01
PRIEST	>0	131398 (94.5%)	99.5	5.6	2.1	99.8
	>1	110351 (79.4%)	97	21	2.4	99.7
	>2	86042(61.9%)	90.4	38.7	2.9	99.5
	>3	65674(47.3%)	82.9	53.5	3.5	99.4
	>4	48134(34.6%)	75.3	66.2	4.3	99.3
	>5	34880(25.1%)	68	75.8	5.4	99.2
	>6	24994(18.0%)	59.3	82.9	6.6	99.
	>7	17400(12.5%)	48.3	88.2	7.7	98.8
	>8	12047(8.7%)	40.3	92	9.3	98.7
	>9	8193(5.9%)	32.1	94.6	10.8	98.6

			Any adverse outcome			
**Tool**	**Threshold**	**N (%) above threshold**	**Sensitivity (%)**	**Specificity (%)**	**Positive Predictive Value (%)**	**Negative Predictive Value (%)**
	>10	5379(3.9%)	24	96.5	12.3	98.4
	>11	3293(2.4%)	15.7	97.9	13.2	98.3
	>12	1970(1.4%)	10	98.8	14	98.2
	>13	1312(0.9%)	7.4	99.2	15.7	98.14
	>14	788(0.6%)	4.8	99.5	16.9	98.09
	>15	459(0.3%)	2.7	99.7	16.6	98.05
	>16	287(0.2%)	1.6	99.8	15	98.03
	>17	153(0.1%)	0.8	99.9	15	98.02
	>18	80(0.1%)	0.5	99.95	16.3	98.01
WHO	>0	78355(56.5%)	73	43.8	2.6	98.8
TEWS	>0	130228 (95.1%)	97.4	5	2	99
	>1	93646(68.4%)	86.6	32	2.5	99.2
	>2	72750(53.1%)	76.5	47.4	2.8	99
	>3	34776(25.4%)	56.8	75.2	4.4	98.9
	>4	19030(13.9%)	41.9	86.7	5.9	98.7
	>5	8581(6.3%)	28.9	94.2	9.1	98.5
	>6	3657(2.7%)	16.4	97.6	12.1	98.3
	>7	1551(1.1%)	8.6	99	15	98.2
	>8	593(0.4%)	4.8	99.7	21.6	98.1
	>9	214(0.2%)	1.8	99.9	22.9	98.06
	>10	90(0.1%)	0.7	99.95	22.2	98.04
	>11	34(0.0%)	0.4	99.98	29.4	98.04
	>12	8(0.0%)	0.1	100	25	98.03
Quick COVID	>0	23300(16.6%)	59.1	84.3	7.1	99
	>1	17203(12.2%)	53.4	88.6	8.7	99
	>2	9865(7.0%)	45.2	93.8	12.8	98.8
	>3	8529(6.1%)	42.4	94.7	13.9	98.8
	>4	6356(4.5%)	39.3	96.2	17.2	98.7
	>5	2574(1.8%)	14.1	98.4	15.2	98.3
	>6	1258(0.9%)	8.2	99.3	18.1	98.2
	>7	389(0.3%)	2.2	99.8	15.4	98.1
	>8	121(0.1%)	1	99.9	24	98

Supplementary Material 4: Flexible calibration curves using locally weighted scatterplot smoothing (LOESS) primary outcome whole study period

Expected versus predicted probabilities plotted by decile groups are compared to a slope of 1 (a perfectly calibrated model). A calibration curve smaller than 1 indicates predicted risks were overestimated for patients at higher risk (shows overfitting).

CRB-65

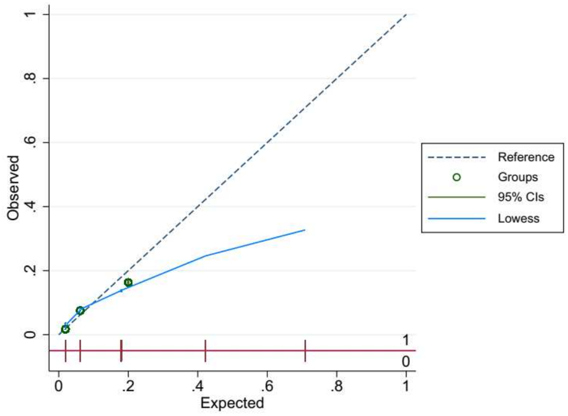

NEWS2

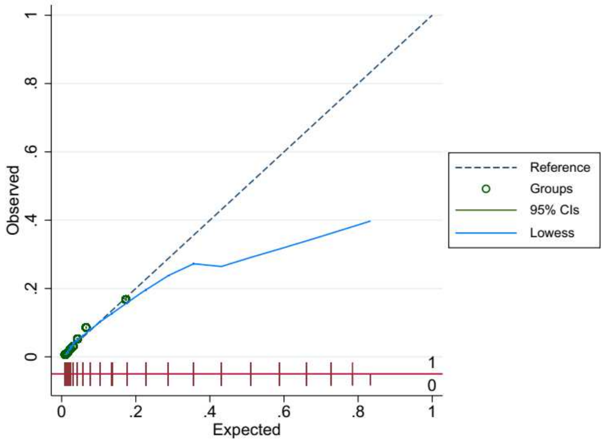

PMEWS

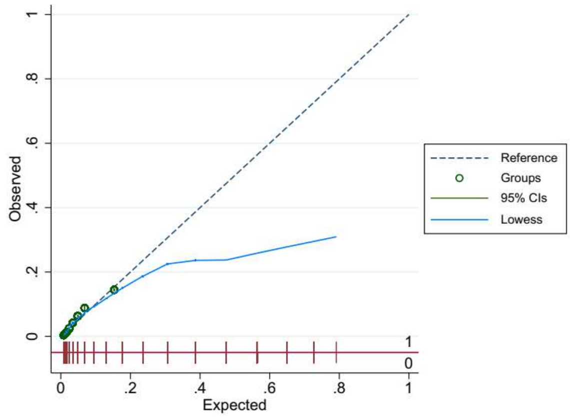

PRIEST

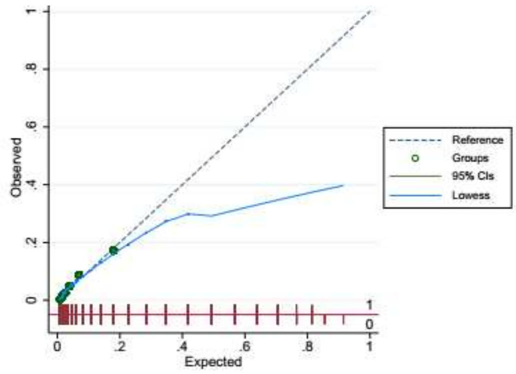

Quick Covid Score

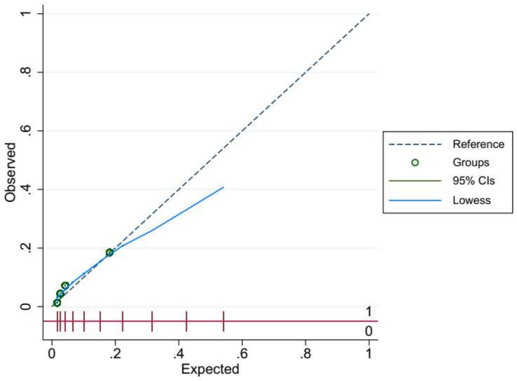

TEWS

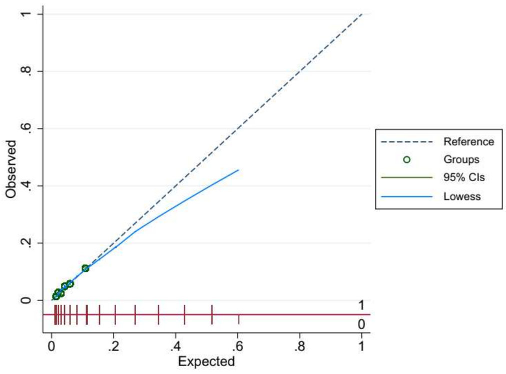

WHO Score

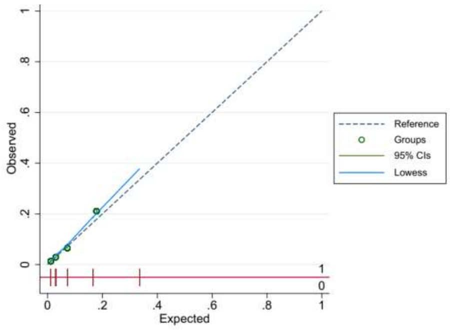

Supplementary Material 5: Performance of tools predicting composite secondary outcome death

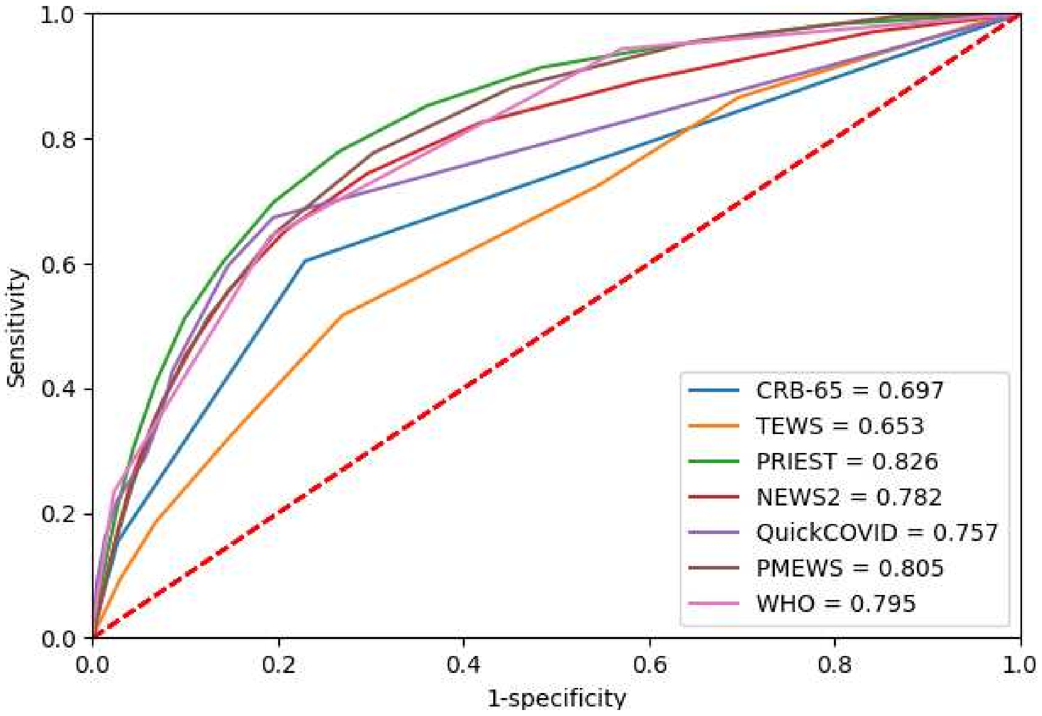

Supplementary Material 6: Performance of tools predicting composite secondary outcome ICU admission

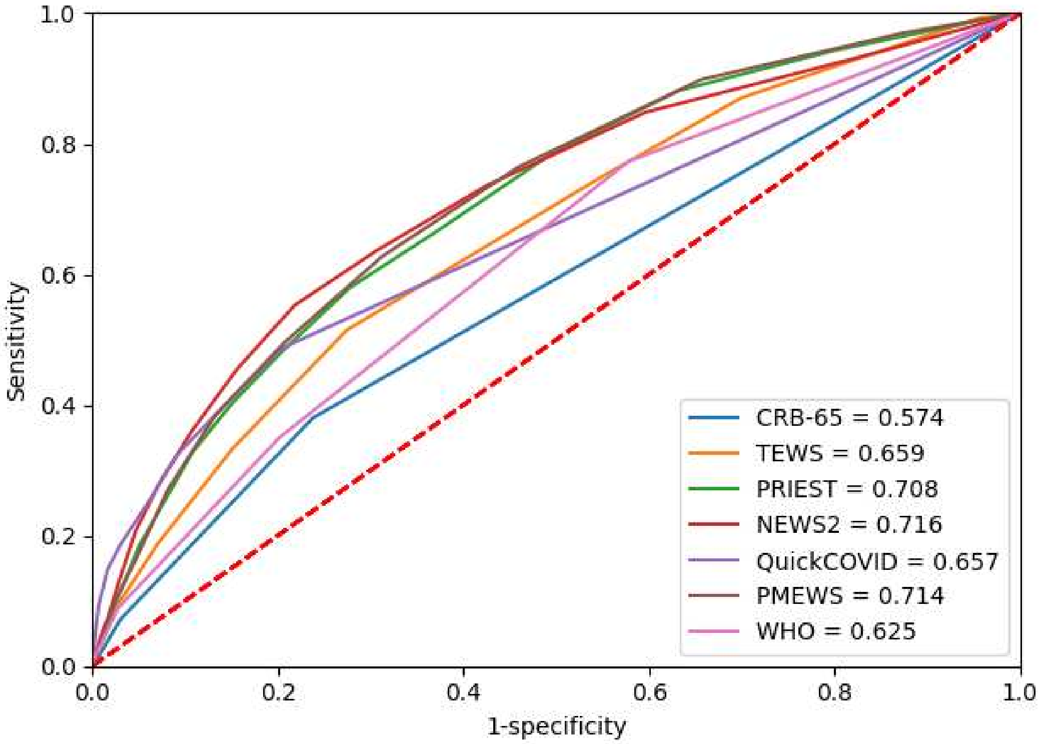

Supplementary Material 7: Probability adverse outcome by score

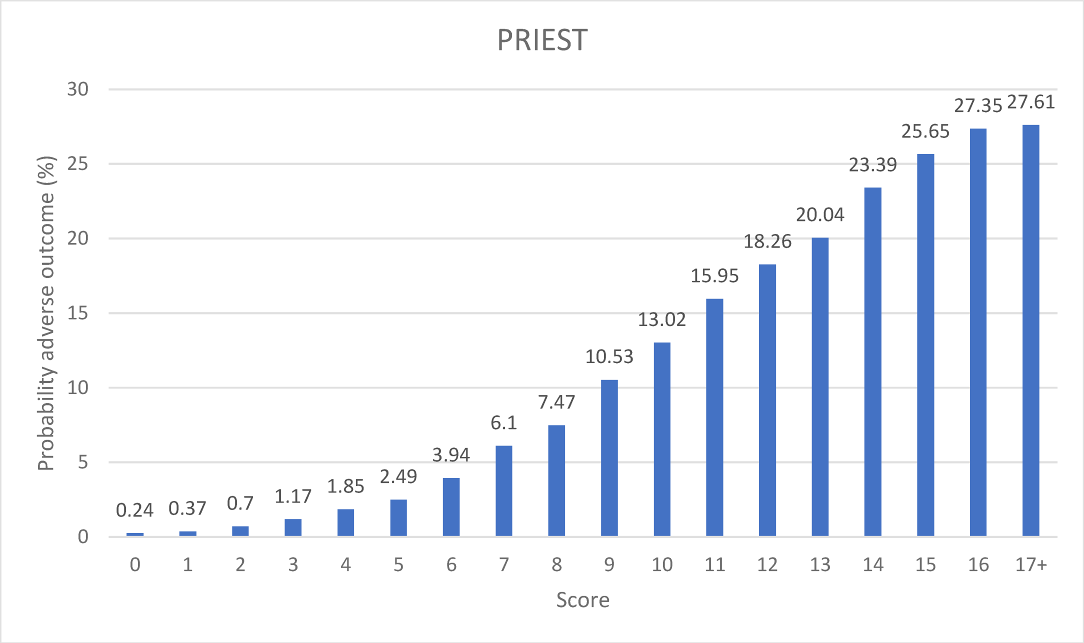
PRIEST
Probability adverse outcome (%)
30
25
20
15
10
5
0
0.24
0.37
0.7
1.17
1.85
2.49
3.94
6.1
7.47
10.53
13.02
15.95
18.26
20.04
23.39
25.65
27.35
27.61
0
1
2
3
4
5
6
7
8
9
10
11
12
13
14
15
16
17+
Score

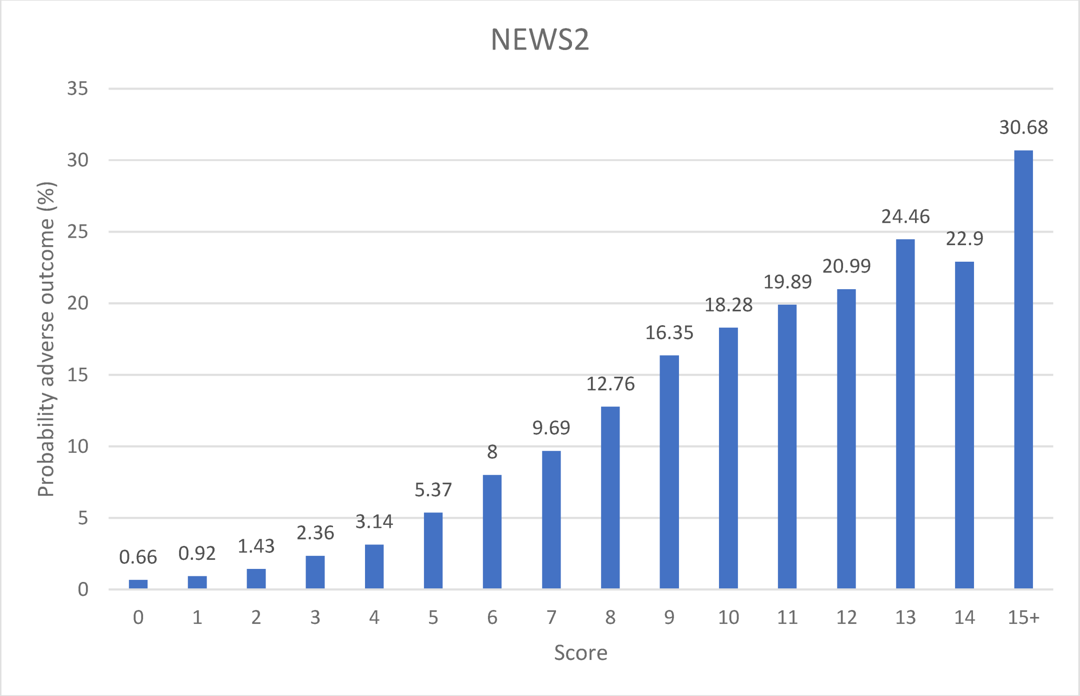
NEWS2
Probability adverse outcome (%)
35
30
25
20
15
10
5
0
0.66
0.92
1.43
2.36
3.14
5.37
8
9.69
12.76
16.35
18.28
19.89
20.99
24.46
22.9
30.68
0
1
2
3
4
5
6
7
8
9
10
11
12
13
14
15+
Score

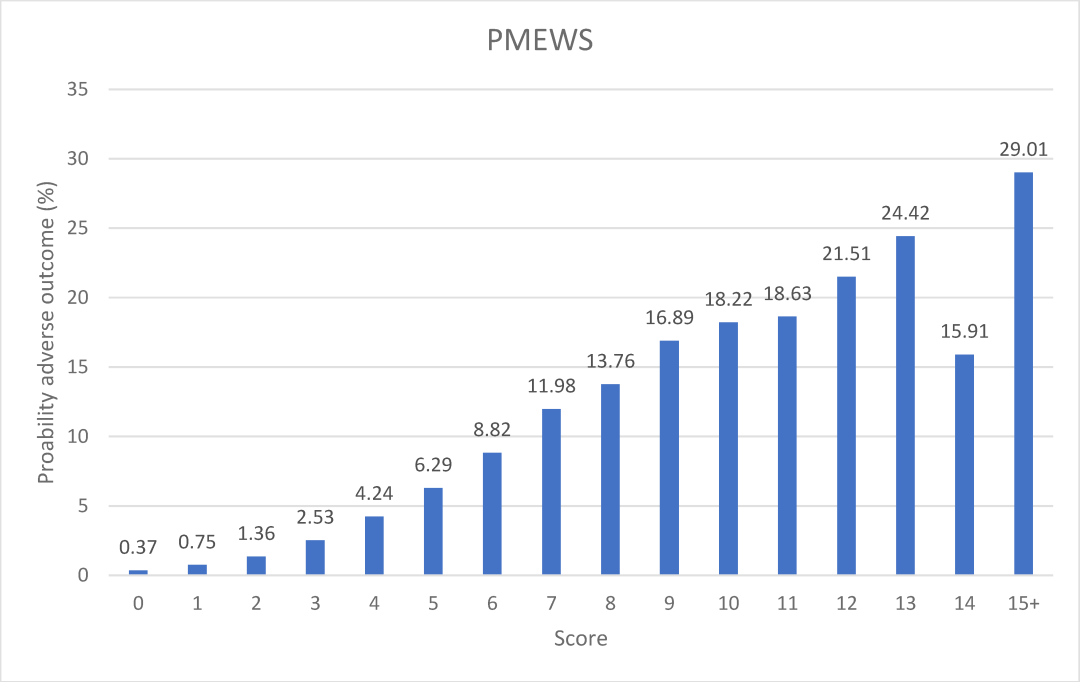
PMEWS
Proability adverse outcome (%)
35
30
25
20
15
10
5
0
0.37
0.75
1.36
2.53
4.24
6.29
8.82
11.98
13.76
16.89
18.22
18.63
21.51
24.42
15.91
29.01
0
1
2
3
4
5
6
7
8
9
10
11
12
13
14
15+
Score
